# The Immune Cellular Effectors of Terrestrial Isopod *Armadillidium vulgare*: Meeting with Their Invaders, *Wolbachia*

**DOI:** 10.1371/journal.pone.0018531

**Published:** 2011-04-20

**Authors:** Frédéric Chevalier, Juline Herbinière-Gaboreau, Joanne Bertaux, Maryline Raimond, Franck Morel, Didier Bouchon, Pierre Grève, Christine Braquart-Varnier

**Affiliations:** 1 Université de Poitiers, Laboratoire Ecologie, Evolution, Symbiose, UMR CNRS 6556, Poitiers, France; 2 Université de Poitiers, Laboratoire Inflammation, Tissus Epitheliaux et Cytokines, EA 4331, Poitiers, France; University of California Merced, United States of America

## Abstract

**Background:**

Most of crustacean immune responses are well described for the aquatic forms whereas almost nothing is known for the isopods that evolved a terrestrial lifestyle. The latter are also infected at a high prevalence with *Wolbachia*, an endosymbiotic bacterium which affects the host immune system, possibly to improve its transmission. In contrast with insect models, the isopod *Armadillidium vulgare* is known to harbor *Wolbachia* inside the haemocytes.

**Methodology/Principal Findings:**

In *A. vulgare* we characterized three haemocyte types (TEM, flow cytometry): the hyaline and semi-granular haemocytes were phagocytes, while semi-granular and granular haemocytes performed encapsulation. They were produced in the haematopoietic organs, from central stem cells, maturing as they moved toward the edge (TEM). In infected individuals, live *Wolbachia* (FISH) colonized 38% of the haemocytes but with low, variable densities (6.45±0.46 *Wolbachia* on average). So far they were not found in hyaline haemocytes (TEM). The haematopoietic organs contained 7.6±0.7×10^3^ *Wolbachia*, both in stem cells and differentiating cells (FISH). While infected and uninfected one-year-old individuals had the same haemocyte density, in infected animals the proportion of granular haemocytes in particular decreased by one third (flow cytometry, Pearson's test = 12 822.98, *df* = 2, *p*<0.001).

**Conclusions/Significance:**

The characteristics of the isopod immune system fell within the range of those known from aquatic crustaceans. The colonization of the haemocytes by *Wolbachia* seemed to stand from the haematopoietic organs, which may act as a reservoir to discharge *Wolbachia* in the haemolymph, a known route for horizontal transfer. *Wolbachia* infection did not affect the haemocyte density, but the quantity of granular haemocytes decreased by one third. This may account for the reduced prophenoloxidase activity observed previously in these animals.

## Introduction

Lacking the memory of vertebrate immunity, invertebrates largely depend upon their innate defensive mechanisms to protect themselves against pathogens and invading organisms. Immune cellular responses include early non-self recognition [1], phagocytosis, cellular encapsulation and nodulation [1]–[3]. Immune humoral responses involve clotting and coagulation reactions [3], [4], the production of antimicrobial peptides [5] and the prophenoloxydase cascade [6]. In crustaceans all these processes are conducted by, or originate from haemocytes which are considered as the cornerstone of their immune system [3], [7], [8].

Most of our knowledge on crustacean immune system stands from decapods, such as freshwater crayfishes, shrimps or crabs which live in aquatic ecosystems. Meanwhile, some isopods (Oniscidae) have evolved a terrestrial lifestyle, which could have impacted their immune system. The latter could thus stand closer to that of other terrestrial arthropods because of similar environmental constraints during their evolution. But for now, the immune system of such terrestrial crustaceans remains poorly described. Concerning the cellular effectors, to this date nothing has been published on the different haemocyte types and their origins. Regarding molecular effectors, only a few papers have been published since 2005 [9]–[12]. In *Armadillidium vulgare*, an antimicrobial peptide acting against Gram positive bacteria was characterized [9] as well as numerous proteins known to be involved in both aspects of the immune response in crustacean decapods and other arthropods [10]. In *Porcellio scaber*, hemocyanin is suggested to fulfil functions of phenoloxidase in addition to serving as a respiratory pigment [11]. In the same species phagocytosis is highly specific upon priming [12], which supports a potentially important role of phagocytes in specific immune responses of invertebrates.

Studying the immunity of terrestrial isopods is also of particular interest to decipher host/symbiont interactions, because they are infected by *Wolbachia* endosymbionts at a high prevalence (62% of terrestrial isopod species are infected [13]). *Wolbachia* are strictly intracellular α-proteobacteria closely related to important human pathogens such as *Rickettsia*, *Ehrlichia*, *Anaplasma* and *Cowdria* [14]. They are maternally inherited symbionts widespread among arthropods and filarial nematodes, probably the most abundant endosymbionts of invertebrates [15], [16]. Such a ubiquity and the wide occurrence of lateral transfers inferred from host/*Wolbachia* comparative phylogenies [17]–[20] suggest that, to improve their own transmission, successful spreading and persistence among host populations, *Wolbachia* are able to avoid and/or to manipulate the host immune system. Indeed, *Wolbachia* manipulate *Aedes albopictus* host antioxidant systems in a manner that is beneficial to its survival [21]. In *Drosophila melanogaster*, *Ae. aegypti* and *Culex pipiens*, *Wolbachia* confer resistance against viruses such as dengue, chikungunya and the West Nile virus but also against the protozoan *Plasmodium* [22]–[26]. In contrast, *Wolbachia* immunodepress *D. simulans* hosts, resulting in less efficient encapsulation of parasitic wasp eggs [27]. In *A. vulgare*, *Wolbachia* infection is associated with immunodepression [28], [29]: the phenoloxidase activity is reduced while the titer of culturable bacteria (i.e. not *Wolbachia*) in the haemolymph increases and the haemocyte density decreases in older specimens.

In this study, we characterized the immune cellular effectors of *A. vulgare* and report for the first time that infected animals, while having normal haemocyte densities, displayed different proportions of haemocyte types. Outstandingly, *A. vulgare* is the only known model system in which *Wolbachia* have been found in haemocytes [28], [30]. We have quantified the extent of such a colonization, and found that the *Wolbachia* were already present in the haematopoietic organs where haemocytes are synthesized and differentiate.

## Results

### Morphological characterization of three haemocyte types

**Figure 1:**
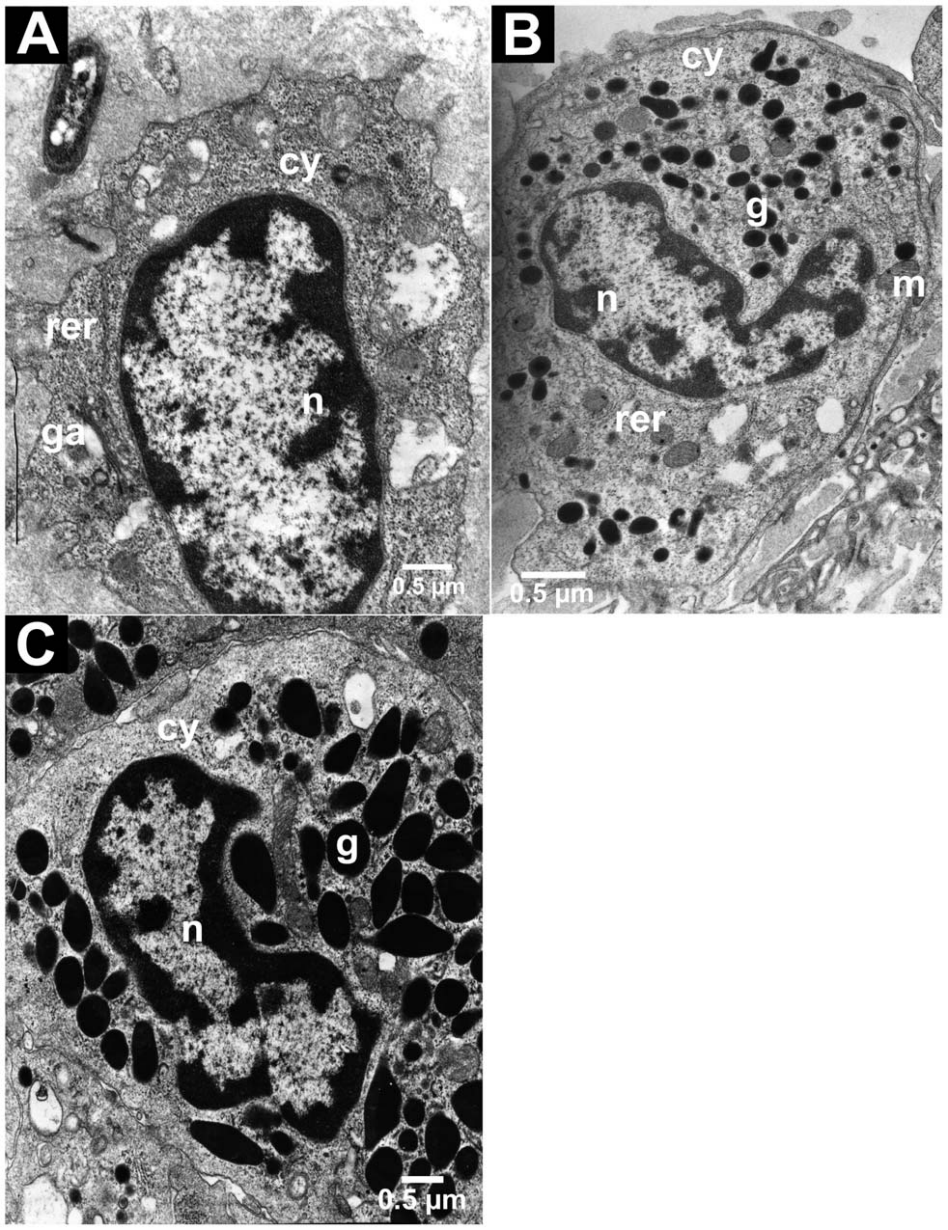
TEM characterization of three haemocyte types. The hyaline type (**A**) did not contain granules, contrary to the semi-granular (**B**) and granular (**C**) types. n: nucleus, cy: cytoplasm, g: granules, m: mitochondria, ga: Golgi apparatus, rer: rough endoplasmic reticulum.

Three haemocyte types were revealed by TEM (Transmission Electron Microscopy): hyaline, semi-granular and granular. Hyaline haemocytes (Fig. 1.A) were relatively small (8 µm×6 µm on average), agranular (or with few granules) and had a high nucleocytoplasmic ratio. The cytoplasm was filled with round electron-dense deposits as well as with rough endoplasmic reticulum (RER), free ribosomes and mitochondria. This haemocyte type represented 7% of the total haemocyte population in the haemolymph (TEM sampled cells: *n* = 58). Semi-granular haemocytes (Fig. 1.B) were larger (12 µm×8 µm on average) and contained abundant, small electron-dense granules (Ø 0.6 to 0.8 µm on average) which presented a homogenous structure. The nucleocytoplasmic ratio was lower than in the hyaline cells. The semi-granular type represented 72% of the total haemocyte population in the haemolymph (TEM sampled cells: *n* = 58). The granular haemocytes (Fig. 1.C) were as large as semi-granular ones but contained abundant, large electron-dense granules (Ø 0.6 to 1.6 µm on average). Golgi apparatus, RER and mitochondria were present in the cytoplasm of both kinds of granular cells. This haemocyte type represented 21% of the total haemocyte population in the haemolymph (TEM sampled cells: *n* = 58).

### Flow cytometry analysis of circulating haemocyte populations of uninfected animals

**Figure 2:**
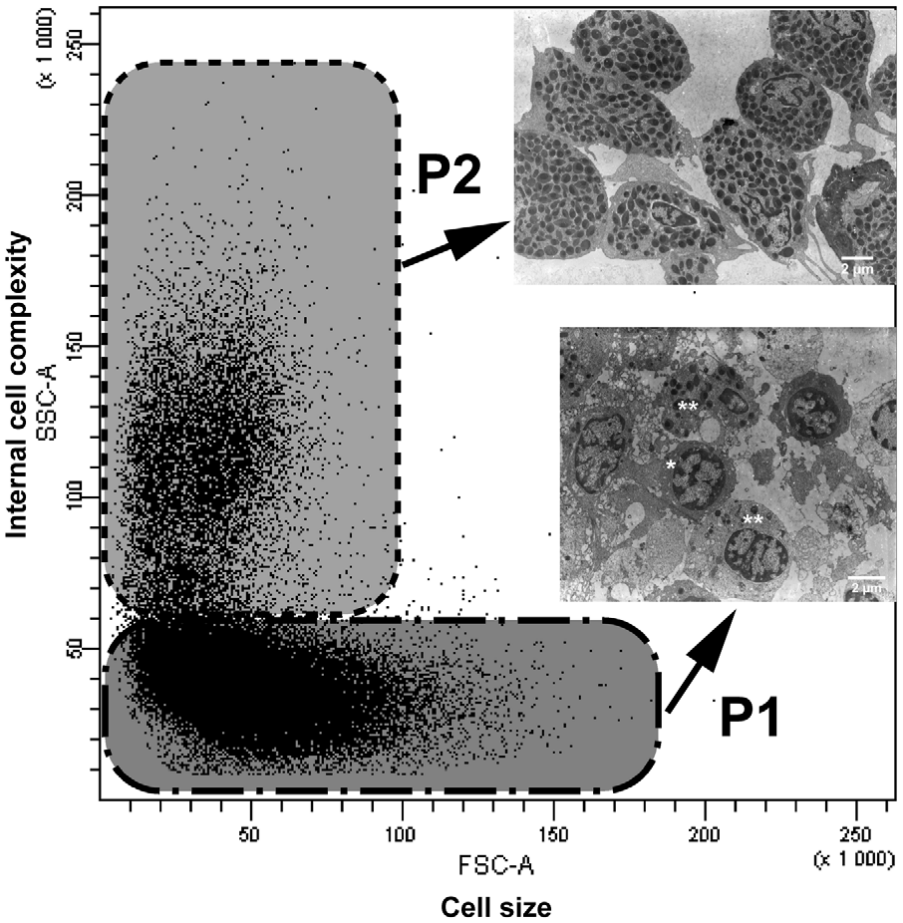
Separation of circulating haemocyte populations. The flow cytometry FSC *vs.* SSC dotplot shows two populations: P1 and P2. After separation on a Percoll gradient, TEM confirmed that P1 contained few hyaline (*) and semi-granular haemocytes (**) and P2 only granular haemocytes. P1 and P2 ellipses drawn manually.

Based on cell size (forward scatter FSC) and internal cell complexity (side scatter SSC), live circulating haemocytes were divided in two populations, accounting for 73% (P1: FSC 4.7±1.2×10^4^, SSC 4.0±5.4×10^4^) and 18% (P2: FSC 4.0±1.1×10^4^, SSC 1.2±0.1×10^5^) of the total circulating haemocytes, respectively (Fig. 2). Dead cells (9%), labelled with propidium iodide, were ignored. The same populations were recovered as two separate bands from gradient centrifugation. From TEM observations, P1 comprised 13% of hyaline haemocytes and 87% of semi-granular haemocytes (sampled cells *n* = 31), while only granular haemocytes were found in P2 (sampled cells *n* = 26) (Fig. 2).

### Assignment of phagocytosis and encapsulation functions

#### *In vivo* phagocytosis experiments

**Figure 3:**
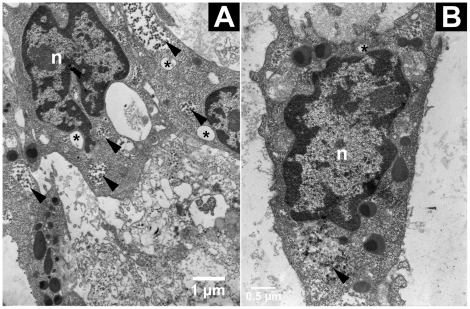
Phagocyted ink particles within lysosomes in haemocytes (TEM). Ink particles were observed in lysosomes (arrowhead) from hyaline (**A**) and semi-granular haemocyte (**B**). n: nucleus, arrowhead: ink particles in a lysosome, asterisk: primary endosome.

In *A. vulgare* ink particles were used to identify phagocytes, which were in majority hyaline haemocytes and in a lower proportion semi-granular haemocytes (Fig. 3). In these cells, numerous lysosomes containing ink particles but also primary endosome could be observed. Ink particles were never found in granular haemocytes.

#### *In vivo* encapsulation experiments

**Figure 4:**
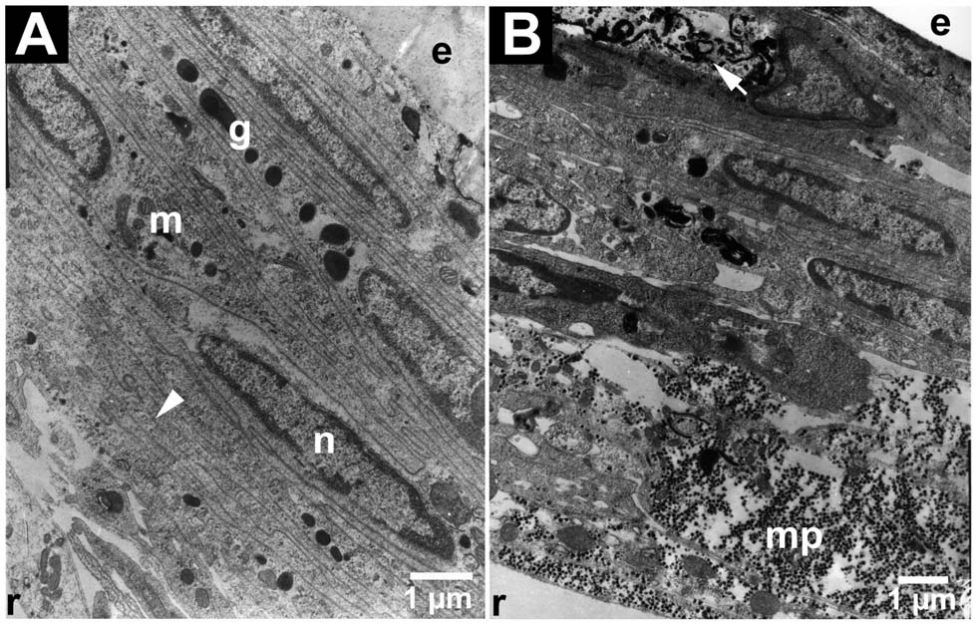
Resin bit encapsulated by haemocytes (TEM). **A**: Semi-granular haemocytes were being recruited at the periphery of the capsule. Some haemocytes in the capsule had lost nucleus and organelles (arrowhead). r: resin side of the capsule, e: external side of the capsule, n: nucleus, g: granules, m: mitochondria. **B**: The capsule was made of several layers of haemocytes, some of them being in an apoptotic stage (arrow). Melanin particles (mp) were released on the resin side.

Resin bits were used to determine the haemocyte type involved in encapsulation. Eight days after implantation, the bits, recovered from the haemocoele, were layered with haemocytes. The capsules resembled a net of strips fitted together, formed by the stretched and joined haemocytes. In the first layers, the cells were much flattened, the nuclei and cytoplasm organelles had disappeared (Fig. 4.A). New haemocytes, not yet stretched, were recruited at the periphery. Semi-granular cells, as per the abundant and small electron-dense granules in their cytoplasm, were the most involved in encapsulation. Melanization products, detected in the haemocyte stacks after eight days (Fig. 4.B), showed the local release of proteins stored in the granules. Some haemocytes presented the formation of myelin bodies and a specific dissociation of the nucleus characteristic of apoptosis (Fig. 4.B).

### Structure of the haematopoietic organs

**Figure 5:**
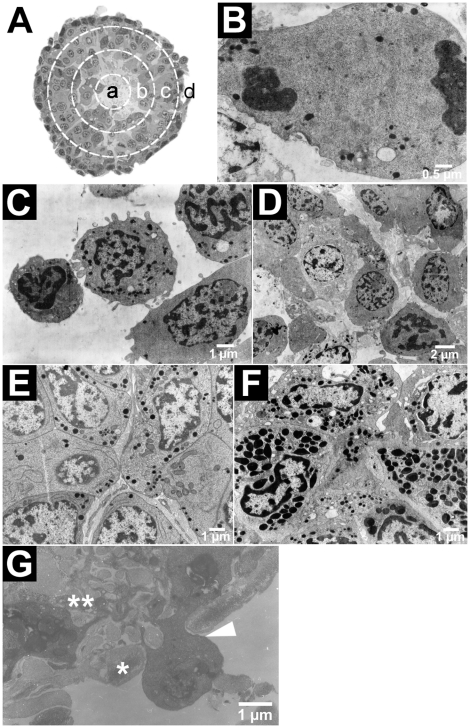
Layout of the haematopoietic organ (TEM). In the haematopoietic organ (**A**, transversal section and schematic layout), the compactness of the tissue and the morphology of the cells allowed discriminating between the central area (a, not visible here but see Fig. 7C), and the internal (b), central (c) and external (d) cortex. The central area contained stem cells (dividing, **B**) isolated and steeped in matrix (**C**, **D**). The granules appeared in the haemocytes from the central cortex on, though the granular types could not be distinguished (**E**). The external cortex contained mostly cells with granules (**F**). A diapedesis figure (arrowhead) across the basal membrane (*) indicated a probable route for haemocyte release from the external cortex (**) into the haemolymph (**G**).

The six haematopoietic organs were 150 µm long, 150 µm large and 50 to 60 µm thick (Fig. 5.A). Each was wrapped in connective tissue limited by a basal membrane. They contained only haemocytes presenting different maturation stages, the least mature being in the central area. There, haemocytes were isolated and steeped in matrix, some had a large nucleus or condensed chromatin, and some were dividing (Fig. 5.B–D). The central area was surrounded by a zone with a lower cell density and then the cortex. The latter was interrupted by irregular ramifications projecting from the low density zone and presented a high cellular density. The cells were larger and their migration to the basal lamina was accompanied by maturation and differentiation. The cortex could be divided into three parts: internal, central and external. In the internal cortex, the cells adhered to each others, were organized and seemed undifferentiated. From the central cortex on, cells with and without granules could be distinguished (Fig. 5.E). Whatever the type, the cell structure was similar: a large nucleus, a reduced cytoplasm, a lot of mitochondria and a well-developed endoplasmic reticulum. In the external cortex, the cells containing granules were more numerous than those without (Fig. 5.F). The two granular haemocyte types could not be discriminated. Diapedesis figures were observed (Fig. 5.G).

### *Wolbachia* infection in haemocytes and haematopoietic organs

**Figure 6:**
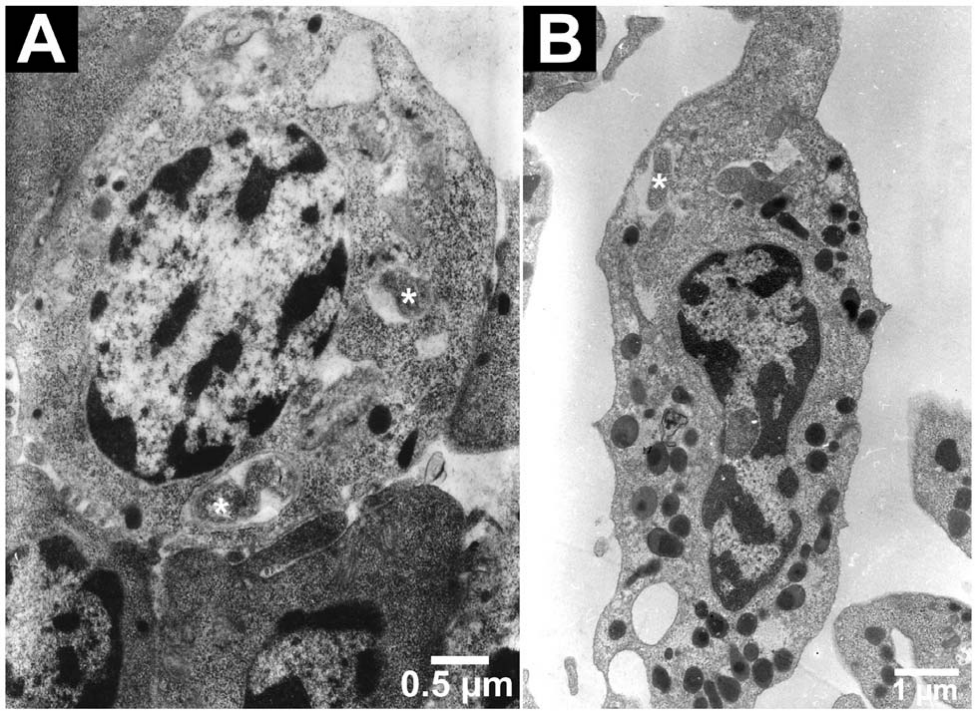
TEM detection of *Wolbachia* in haematopoietic organs and circulating haemocytes. *Wolbachia* (*) in a non-differentiated cell in an haematopoietic organ (**A**) and in a semi-granular haemocyte (**B**).

**Figure 7:**
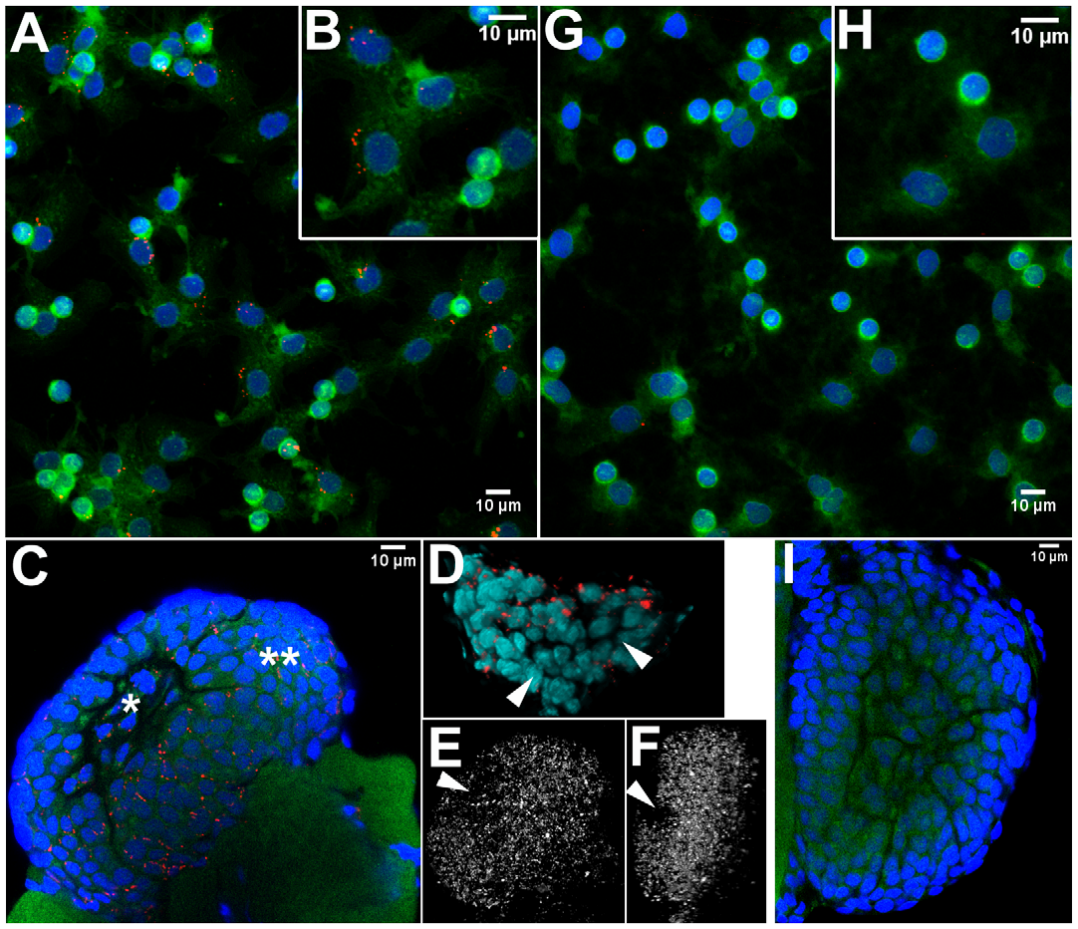
FISH detection of *Wolbachia* in circulating haemocytes and haematopoietic organs. In infected animals, *Wolbachia* (in red) colonized many haemocytes (**A**, **B**) and the central area (*) as well as the cortex (**) of the haematopoietic organ (**C**), although groups of cells remained uncolonized (Arrowheads, **D**–**F**). The control uninfected animals presented only rare *Wolbachia*-like artefacts (haemocytes, **G–H**, haematopoietic organ, **I**). **A**–**C, G–H**: red: *Wolbachia*, green: Actin; blue: Nuclei. **A**, **B**, **G**, **H**: average intensity Z-projections. **B** and **H**: Close-ups. **D**–**F**: 3D analysis (ImageJ 3D viewer) of image **C**. **D**: tilted volume rendering of *Wolbachia* (red) and the nuclei (turquoise) in the central area extracted from the Z-stack. **E** and **F**: volume rendering of *Wolbachia* (grey-scale) from the whole haematopoietic organ. **E**: front view corresponding to image **C**, **F**: tilted view (180°).

*Wolbachia* were observed by TEM in haematopoietic organ cells with granules, and in granular and semi-granular circulating haemocytes (Fig. 6). The bacteria were included in a vacuole and did not seem to be undergoing any type of degradation process. Their 16S rRNA were detectable by FISH (Fluorescence *in situ* Hybridization) (Fig. 7.A–F).

In the five infected animals (Fig. 7.A–B) 35 to 53% of haemocytes were FISH positive. The mean infection rate was 42% (*n* = 1 002 haemocytes). Enumerating the *Wolbachia* themselves was not possible because they often clustered together: instead we measured the cumulated volumes of their fluorescence. The fluorescence of a non-clustering coccoid *Wolbachia* was 0.88 µm^3^ (95% confidence interval: [0.73–1.06]) with no variation between sampled females (ANOVA *F_2,37_* = 1.36, *p* = 0.2678), therefore volumes in µm^3^ are approximately equivalent to *Wolbachia* numbers. Based on this estimate, colonized haemocytes contained 6.45±0.46 *Wolbachia* on average (range 0.3–24.5, in a subsample of 144 haemocytes). In uninfected animals, fluorescent objects that could be mistaken for *Wolbachia* were observed in 4% of the haemocytes (*n* = 723, Fig. 7.G–H). The volume of these artefacts was 2.65±0.41 µm^3^ and was thus much lower than the *Wolbachia* signals (BoxCox transformed data, *t* = −4.20, *df* = 169, *p*<0.001). Since similar artefacts could have contributed to the *Wolbachia* counts, a better estimation of *Wolbachia* haemocyte colonization, excluding artefacts, would be 38%.

The haematopoietic organ nodules contained 7.6±0.7×10^3^ *Wolbachia*, as estimated from their fluorescence volume (Fig. 7.C). The resolution was not sufficient to discriminate the cell borders and enumerate the bacteria in each cell, but it was possible to distinguish the central area from the cortex thanks to the gap in between. The proportion of colonized area was 1% in both parts (Arcsine square-root transformed data ANOVA: zone effect: *F*_1,19_ = 0.8771, *p* = 0.36; infection status effect: *F*_1,19_ = 268, *p*<0.001; interaction infection status×zone: *F*_1,19_ = 0.0009, *p* = 0.9759). Not all the cells were colonized, in both zones (Fig. 7.D–F): some areas of several neighbouring cells were devoid of bacteria. The number of *Wolbachia* per cell seemed extremely variable. Very occasionally, they clustered in high numbers around the nucleus in haemocytes from the cortex periphery. The background fluorescence recorded from uninfected animals (Fig. 7.I) was comparatively low (whole organ: 0.1±0.05×10^3^; central area: 0.002±0.002×10^3^; cortex: 0.10±0.05×10^3^ µm^3^).

### Perturbation of haemocyte proportions in *Wolbachia*-infected animals

In one-year old infected animals, the mean number of circulating haemocytes (2.1±0.4×10^4^ cells/µl haemolymph; *n* = 14) was the same as the one observed in uninfected animals of the same age (3.2±0.4×10^4^ cells/µl haemolymph; *n* = 14, *t* = −1.88, *df* = 26, *p* = 0.0804). The haemocytes of populations P1 (FSC 4.7±1.2×10^4^, SSC 4.0±0.5×10^4^) and P2 (FSC 4.0±1.1×10^4^, SSC 2.0±1.6×10^4^) resembled those of uninfected animals in internal complexity and size (P1-FSC: *t* = 0.12, *df* = 25, *p* = 0.9039; P2-FSC: *t* = −0.34, *df* = 25, *p* = 0.7373; P1-SSC: *t* = −1.44, *df* = 25, *p* = 0.1628; P2-SSC: Wilcoxon's test = 0.54, *df* = 1, *p* = 0.4642) but the proportion of the populations differed (Pearson's test = 12 822.98, *df* = 2, *p*<0.001), with 12% of granular haemocytes (P2), 76% of hyaline and semi-granular haemocytes (P1) and 12% of dead cells.

## Discussion

### The immune cellular effectors of *Armadillidium vulgare*…

In invertebrates, circulating haemocytes are central to the innate immune system, being involved in phagocytosis and encapsulation. They are also vehicles for other immune functions such as the generation of reactive oxygen and nitrogen species, as well as the production of antimicrobial peptides and enzymes involved in the phenoloxydase (PO) cascade [1], [31].

In most crustacean species, the haemocyte classification is based on the presence/absence of cytoplasmic granules. Following this methodology, three types of circulating haemocytes are usually recognized [7]: hyaline haemocytes without evident granules and a high nucleo-cytoplasmic ratio, semi-granular haemocytes harbouring a variable number of small granules in their cytoplasm, and granular haemocytes with numerous large granules. In the terrestrial isopod *Armadillidium vulgare*, we clearly observed these three types of haemocytes as described in shrimps [32], [33], in freshwater crayfishes and in crabs [34], [35].

Each haemocyte type is thought to have a dominating function. Because the cytoplasmic granules of crustacean haemocytes contain the humoral proteins (agglutinins, peroxinectins, enzymes of the coagulation and the PO cascade, antimicrobial peptides [36]), their presence is easily associated with coagulation and encapsulation. Phagocytosis is trickier to assign. In shrimps, semi-granular and granular haemocytes are able to phagocyte yeast particles *in vitro*, but not hyaline haemocytes [33], [37]. In contrast, only the hyaline haemocytes can phagocyte latex beads *in vitro* [38]. In the freshwater crayfish, all haemocyte types show some phagocytic response but only the semi-granular ones are involved in the phagocytosis of all foreign particles used in the *in vivo* assay [39]. In *A. vulgare*, the China ink particles were phagocyted by the hyaline haemocytes and some semi-granular haemocytes. The resin bits were encapsulated by semi-granular and granular haemocytes. The latter formed multiple layers across the resin, stretching themselves. Some had lost their nucleus or were apoptotic: such cellular lyses might drive the release of the granule content, especially the enzymes of the melanization cascade and the agglutination proteins like in other crustaceans [40], [41]. Congruently, some melanization phenomena were detected.

As in other invertebrates, crustacean haemocytes are produced in specialized tissues: the haematopoietic organs. Each lobule is surrounded by connective tissue and contains stem cells, differentiating haemocytes, and mature blood cells. Haematopoietic organ location varies even between close taxa. In lobsters, crabs or freshwater crayfishes, the haematopoietic tissue is composed of a series of ovoid lobules that collectively form a thin sheet on the dorsal surface of the foregut [8]. In the shrimp *Sicyonia ingetis*, the haematopoietic organs occur as a pair of nodules on the dorsolateral surface of the foregut [8]. The haematopoietic organs of *A. vulgare* were composed of three pairs of nodules, localized in the sixth and seventh abdominal segments and in the first telson segment against the pericardia septum as described previously in another isopod species (*Porcellio dilatatus*) [42]. Each nodule was wrapped in connective tissue which was limited by a basal membrane. Each contained haemocytes at different maturation stages, the least mature being in the central area. In crustaceans, it is still unknown whether granular and hyaline haemocytes stem from the same cells [36], and whether they follow a single line or two separate lines of differentiation. It is believed that maturation is complete when the haemocytes are released into the circulation [3], but the mechanisms behind this release are still unknown, as in most invertebrates. In *A. vulgare*, we observed diapedesis figures suggesting that haemocytes are released at least this way in this species.

### … Meeting with their invaders, *Wolbachia*

When *Wolbachia* infect insect somatic tissues, they can be found in the haemolymph [43]–[46] though not always [47]–[49]. Their presence is revealed through PCR detection, or upon transfection experiments (the haemolymph proves infectious) [43]–[46]. They are believed to remain extracellular in the plasma [50], especially since Rasgon *et al.* [51] showed they can survive outside cells. *Wolbachia* are observed inside haemocytes only in the crustacean *A. vulgare* [28], [30]. Their identification stands from their morphology (TEM) and from specific PCR assays (*wsp* gene, data not shown; type IV secretion system genes, [52]). They are enclosed in vacuoles, though TEM observations reveal no degradation patterns. Here we further confirmed they were alive by the FISH labelling of their 16S rRNA [53] using a *Wolbachia*-specific probe [54]. Haemocyte colonization is not fortuitous insofar that it has been routinely observed over the years [28], [30], plus we showed here that more than one third of the haemocytes was colonized.

The presence of live *Wolbachia* within haemocytes, the main actors of the immune system in crustaceans, can be surprising. Actually, *Wolbachia* are successfully transfected inside insect haemocyte-like cell lines, responding to bacterial challenge by immune responses such as phagocytosis or antimicrobial peptides synthesis [51], [55]. But the stability of such transfections varies from a massive colonization to stabilization at 10% and even elimination [51], [55]. It is suspected that the activation of some immune functions could limit *Wolbachia* invasion [55]. Here, in a host that inherited *Wolbachia* naturally through maternal transmission, haemocyte colonization was not massive (38% prevalence, six *Wolbachia* per cell on average). Notably so far TEM observations never revealed *Wolbachia* in hyaline haemocytes, suggesting that they could escape (lacking the appropriate surface receptors?) or resist infection (by destroying *Wolbachia*?). To investigate this, we need to develop fluorescent markers for each cell type, compatible with FISH. *Wolbachia* could ultimately serve as a marker of the granular and semi-granular cell lineages.

Regarding the origin of *Wolbachia* in haemocytes, it is doubtful that they were acquired from the plasma, since in our case they are not detected there by PCR (personal observation). Rather, the colonization would stand from the infection of the haematopoietic organs which was reported here for the first time. The *Wolbachia* were found in the stem cells as well as the differentiating cells until the very edge of the organs, suggesting that infected haemocytes can be released in the haemolymph. Not all the cells were infected: this could simply result from stochastic loss during mitosis. Uninfected cells also happened as clusters, although it was impossible to tell whether they radiated from an uninfected stem cell, be it hyaline ones or other. Since we could not enumerate the cells in the haematopoietic organs, we cannot link their colonization status with that of the haemocytes. Still the *Wolbachia* load was conserved across the central area and the cortex.

*Wolbachia* are detected in many somatic tissues in *A. vulgare*, but the colonization of the haematopoietic organs in particular may have further implications. Rigaud *et al.* [30] proposed the haemocytes to shuttle *Wolbachia* across the organism to infect or re-infect tissues. It makes sense insofar that transfection by short blood contact proved haemolymph to be infectious [56]. Also Rigaud *et al.* [30] found the colonized haemocytes in the vicinity of the oocytes during re-colonization after temperature curing. We propose that the haematopoietic organs act as a reservoir, similar to the Somatic Stem Cell Niche described by Frydman *et al.* [57] in *Drosophila*, although part of their argument is that cells there seldom divide and can therefore accumulate *Wolbachia*. Here, more than a reservoir it would be a factory where *Wolbachia* are packed safely inside cells, and delivered systematically in the haemolymph along with the haemocytes, throughout the life of the host, ensuring their location at the right place for horizontal transmission.

Animals investigated in this study were one-year-old and they had the same haemocyte density whether they were infected or not by *Wolbachia*. However, strikingly they exhibited different proportions in the haemocyte populations we were able to separate. In infected animals, the percentage of hyaline and semi-granular haemocytes only very slightly increased, while granular haemocytes decreased by one third. In crustaceans, the PO cascade is stored in the granular haemocytes [8]: we have shown earlier that the PO activity is also reduced by one third in the very same animals [29]. In two-year-old infected animals the reduction in PO activity is even stronger, this time with a decrease in haemocyte densities [28], [29]: with FACS, we will now confirm whether only granular haemocytes are affected. This would highlight that the presence of *Wolbachia* impairs an immune function through a cell type.

To sum up, we have characterized for the first time the immune cells of a terrestrial isopod, which turn out to resemble those of a shrimp. The potential diapedesis figures observed in the haematopoietic organs require further investigation, since it is the first clue for a mechanism of haemocyte release in crustaceans. In full opposition with what is known so far in insects, the endosymbionts *Wolbachia* colonized one third of the haemocytes as well as the haematopoietic organs. In infected animals the density of granular haemocytes decreased, which may account for the functional deficiencies we had observed. The question remains whether it presents any advantage for *Wolbachia*.

## Methods

### Ethics statement

All experimental procedures and animal manipulations did not require an ethics statement.

### Animals

*Armadillidium vulgare* individuals infected by a feminizing *Wolbachia* strain (*w*VulC) [30], [58] (originating from Helsingör, Denmark or Celles-sur-Belle, France) or uninfected (originating from Helsingör, Denmark or Nice, France [17], [29]), were investigated. As *A. vulgare* males are never infected by *Wolbachia*, females only were used in this study. They were taken from laboratory lineages sampled 20 years ago from natural populations, reared at 20°C under natural photoperiod with food provided on an *ad libitum* basis. Flow cytometry experiments were realized on one-year old animals, the same age as in Sicard *et al.* [29].

### Haemolymph and haematopoietic organs sampling

Cuticles were disinfected by immersing individuals for 30 s in a 10% sodium hydrochloride solution followed by a 30 s immersion in distilled water. The cuticle of animals was pierced dorsally between the sixth and the seventh dorsal abdominal segments using a needle and haemolymph was collected with a micropipette. The haematopoietic organs were dissected. As in the majority of terrestrial isopods, the three pairs are localized between the sixth and seventh abdominal segment and the first telson segment, along the dorsal vessel [42].

### *In vivo* phagocytosis and encapsulation experiments

Phagocytosis phenomena were investigated by injecting China ink particles (Ø 30–40 nm; Pelikan, Günter Wagner) into animals. After washing in bidistilled water, the China ink particles were suspended in Ringer buffer (1.4 mM CaCl_2_, 2.4 mM HNaCO_3_, 2 mM KCl, 0.4 M NaCl). One microlitre was injected into the general cavity of *A. vulgare*. After two days, haemocytes were recovered from haemolymph and treated for Transmission electron microscopy (TEM) observations according to Braquart-Varnier *et al.* [28].

To investigate encapsulation phenomena, small resin cylinders (5 mm long) were introduced under the cuticle of animals. Eight days after implantation, the animals were dissected and the resin bits were fixed. For scanning electron microscopy, samples were treated as for TEM [28].

### Fluorescence *in situ* hybridization (FISH)

Haemocytes and haematopoietic organs were sampled from five females. Two microlitres of haemolymph per individual were spotted on a polylysine coated slide (Kindler GmbH & Co., Germany) in a well (Ø∼0.7 mm) drawn with a liquid repellent pen (Daido Sangyo Co. Ltd, Japan) and covered immediately with a 18×18 mm coverslip to prevent evaporation. The haemocytes were allowed to spread for 30 min at 4°C in a humid chamber. The slides were immersed for 5 min in a 1% paraformaldehyde-PBS solution (137 mM NaCl, 8 mM Na_2_HPO_4_, 12H_2_O, 1.5 mM KH_2_PO_4_, 3 mM KCl, pH 7.3). To discard the autofluorescent proteins of the haemolymph, the coverslips were shaken down immediately. The slides were washed in PBS for 5 min, dehydrated in 50, 80, 96% ethanol baths for 3 min each. The samples were hybridized according to Manz *et al.* [59] modified, in 20 µL of hybridization buffer per well, containing 35% formamide, Triton X-100 replacing SDS, 2 µL of an equimolar mixture of probes W1,2-Cy3 (30 ng.µL^−1^) specifically targeting *Wolbachia* 16S rRNA [54] and 0.2 µL FITC-phalloïdin (Sigma, dried from methanol 100 µg.mL^−1^ stock solution) targeting actin to show cell outlines. After washing and drying the samples, they were mounted in a mixture of DAPI (2.5 µg.mL^−1^, Sigma) to label the nuclei and Citifluor (AF1 antifading, Citifluor, England). The haematopoietic organs were treated similarly, but due to their small size and because they did not attach to the slides, all the solutions were pipetted on the wells. They were fixed for 1 h in 20 µL of 3% paraformaldehyde-PBS, washed twice in PBS for 15 min and dehydrated.

Detection was performed with an Olympus confocal laser scanning microscope (Olympus IX81) and FV1000 2.0 software (Olympus) equipped with a 60× objective (PLAPO, water immersion, 1.2 NA), with Helium Neon (543 nm, to detect W1,2-Cy3), Argon (488 nm, for phalloïdin) laser lines and a blue diode (405 nm, for DAPI). The images were recorded in sequential mode, with 0.5 µm steps between slices to scan the whole volume of the organ or cells. The voxel size was 0.265×0.265×0.5 µm^3^.

The analyses were performed with Image J 1.42q [60]. The number of haemocytes and their *Wolbachia* colonization status were recorded in five random images (212×212 µm^2^) per female. Haemocytes touching the image borders, i.e. incomplete ones, were ignored. The *Wolbachia* titers in cells were estimated in 144 haemocytes (eight photos from three animals) and one haematopoietic organ of each of the five females. The volume of *Wolbachia* fluorescence was measured semi-automatically by selecting the bacteria within a Region Of Interest (ROI), thresholding automatically each image in the stack and applying the “Voxel counter” plugin. Five non-clustering *Wolbachia* were chosen in each photo (*n* = 40) to estimate the mean volume of one bacterium. The volumes measured can subsequently be converted to equivalent *Wolbachia* numbers. To infer the amount of artefacts, the same analyses were run on uninfected animals: the proportion of haemocytes containing signals resembling *Wolbachia* and the corresponding autofluorescent voxels were enumerated (27 haemocytes, 15 photos from three animals). Images from infected and uninfected animals were treated with the same parameters from the acquisition on.

### Separation of haemocyte populations

Continuous gradients of 9 mL of 55% Percoll (GE Healthcare, Uppsala, Sweden) in Ringer isopod solution (1.4 mM CaCl_2_, 2.4 mM NaHCO_3_, 2 mM KCl, 0.4 M NaCl) adjusted to 0.194 M NaCl were preformed (28 000×g, 30 min, 4°C). To minimize cell attachment to the centrifuge tube wall, all tubes were washed in 6 M urea, pH 2 and rinsed thoroughly in distilled water before use [34].

The haemolymph from 30 animals was collected on ice and half diluted in an anticoagulant buffer (MAS: 9 mM EDTA; 115 mM glucose; 336 mM NaCl; 27 mM sodium citrate, pH 7 [61]). Six hundred microlitres of this haemolymphatic solution were added on the top of the Percoll gradient and centrifuged (400×g, 20 min, 15°C). Cell bands were collected individually and one volume of Ringer isopod solution was added to break the gradient. The haemocytes were pelleted (200×g, 15 min, 4°C) and resuspended in the appropriate solution either for TEM or for flow cytometry analyses.

### Flow cytometry

The haemocytes were suspended in MAS buffer, enumerated with an automated Cell Counter (Invitrogen Countess™) and the cell titer was adjusted to 10^5^ haemocytes per 500 µL.

Flow cytometry was performed with a FACS Canto II (BD Biosciences) equipped with Argon (488 nm) laser. Dead cells were labelled with propidium iodide (final concentration 5 µg.mL^−1^) to exclude them. Two cytogram parameters based on forward scatter height (FSC) and side scatter height (SSC) of unlabelled viable cells were designed. For each haemocyte sample, 50 000 events were counted. The results were expressed as a dot plot indicating the cell size (FSC value) and the internal cell complexity (SSC value). Diva 6.0 software (BD Biosciences) was used to create logical regions.

### Statistical analyses

All statistical analyses were performed using JMP v2.5 software (SAS Institute Inc., Cary, NC, USA). Percentage estimates were arcsine-square-root transformed to meet homogeneity of variances and normality. Measures of the volume of fluorescence were analysed after Box–Cox transformation [62]. The FSC and SSC values were checked for normal distribution (Shapiro-Wilk's test) and homoscedasticity (Bartlett's test). When the data fitted the normal distribution, a *t* test was performed. Otherwise Wilcoxon's test was used. The numbers of events were compared with Pearson's tests.
